# ﻿ *Aster xuelinii* (Astereae, Asteraceae), a new species growing on moist rocks from Gansu Province, China

**DOI:** 10.3897/phytokeys.263.166400

**Published:** 2025-09-15

**Authors:** Zengfu Bai, Zhihua Zhang, Guojin Zhang, Ji Zhang

**Affiliations:** 1 College of Life Sciences, Northwest Normal University, Lanzhou, 730070, Gansu, China; 2 Institute of New Rural Development, Northwest Normal University, Lanzhou, 730070, Gansu, China; 3 College of Life Sciences, Hunan Normal University, Changsha, Hunan, 410081, China

**Keywords:** *
Aster
*, Gansu, molecular phylogeny, morphological characters, new taxon

## Abstract

*Aster xuelinii*, a new species from Gansu Province, China, is described and illustrated based on morphological and phylogenetic evidence. The new species is superficially similar to *A. dolichopodus* Y. Ling in that both have nearly entire leaves, synflorescence solitary at branch tips or arranged in corymbiform clusters, and ray floret color. However, it differs from *A. dolichopodus* in having peduncles 6–9 cm in length and 12–14 ray florets, versus 2.5–15 cm in length and 19–26 ray florets. Phylogenetic analyses of nuclear ribosomal ITS and ETS sequences, as well as the chloroplast *trnL-F* region, place this new species within the genus *Aster*, where it forms a well-supported clade with *A. taliangshanensis* Y. Ling. *A. xuelinii* differs from *A. taliangshanensis* in that the latter has stems often purplish-red in the upper part, capitula typically arranged 1–3 at branch tips forming a loosely corymbiform synflorescence, and a higher number of ray florets (50–60), which are bluish-purple. Furthermore, detailed morphological descriptions, diagnostic illustrations, and ecological habitat characteristics are provided, supporting the classification of this new species.

## ﻿Introduction

*Aster*Linnaeus (1753: 872), the type genus of Asteraceae, comprises approximately 187 species (POWO 2025) and is widely distributed across Asia, Europe, and North America (Chen et al. 2011). Plants of this genus occupy diverse habitats, from low-altitude ravines and river valleys to mid-elevation forest edges and meadows, extending up to high-altitude alpine meadows, scree slopes, and glacial zones. This broad distribution demonstrates their remarkable ecological adaptability.

China harbors over 170 *Aster* species, including taxa formerly classified in segregate genera such as *Heteropappus* Less. and *Doellingeria* Nees (Li and Liu 2002; Li and Zhang 2004; Li et al. 2017, 2020, 2021; Xiao et al. 2019a, b; Xiong et al. 2019; Zhang et al. 2015, 2019). Modern studies identify the Qinghai–Tibet Plateau and Yunnan–Guizhou Plateau as the primary centers of diversity for the genus (Xiao et al. 2019a).

During 2024 fieldwork in Gansu Province, an undescribed *Aster* species was collected at a damp wall close to a river. Following morphological examination and phylogenetic analyses, we confirm its status as a novel taxon, naming it *Aster xuelinii*, and provide a detailed description with comprehensive diagnostic characters herein. As demonstrated by Zhang et al. (2019), Mitsui et al. (2011), and Mitsui and Setoguchi (2012), streamside-adapted species frequently exhibit narrow leaves – a morphological adaptation that minimizes mechanical stress from flowing water.

## ﻿Materials and methods

### ﻿Morphological observations

Morphological data were collected during field observations conducted in Xiaolongshan National Nature Reserve, Hui County, Gansu Province. Voucher specimens were photographed with a Nikon D750 digital camera and deposited in the Herbarium of the College of Life Sciences, Northwest Normal University (NWTC) (https://laifu.nwnu.edu.cn/3873/list.htm). The description of the new species *Aster xuelinii* was conducted using stereomicroscopy on both fresh and dried specimens, with all measurements based exclusively on living material. A comparative analysis was conducted between the target species and its morphologically similar congenerics, particularly *A. dolichopodus*, to delineate diagnostic characteristics.

### ﻿Molecular systematics

#### ﻿Taxon sampling

Molecular phylogenetic analyses were conducted using sequences from three regions: ITS, ETS (White et al. 1990; Baldwin and Markos 1998; Linder et al. 2000; Stanford et al. 2000; Markos and Baldwin 2001), and *trnL-F* (Taberlet et al. 1991). The study included 16 taxa, with *Chrysanthemum indicum* L. and *Callistephus chinensis* (L.) Nees designated as outgroups following previous studies (Li et al. 2012; Zhang et al. 2015). Sequences for 14 species were retrieved from GenBank. Voucher specimens for newly sequenced samples are deposited in the NWTC Herbarium. Voucher information and GenBank accession numbers are provided in Suppl. material 1.

### ﻿DNA extraction, ampliﬁcation, and sequencing

Leaf tissues were collected in the field and dried in silica gel. DNA extraction, purification, and sequencing followed the protocol described by Zhang et al. (2015). For PCR amplification, ITS and ETS regions were amplified using the methods of Linder et al. (2000), while the *trnL-F* region followed Zhang et al. (2015). Specific primers employed were ITS primers from Linder et al. (2000); ETS primers “Ast-8” (Markos and Baldwin 2001) and “18S-IGS” (Baldwin and Markos 1998); and *trnL-F* primers “c” and “f” (Taberlet et al. 1991).

### ﻿Phylogenetic analysis

Sequence alignments were performed using the MAFFT online version (Katoh et al. 2017), followed by manual adjustment in BioEdit v7.2 (Hall 1999). The optimal DNA substitution models were selected under the Akaike Information Criterion (AIC) using jModelTest v2.1.7 (Darriba et al. 2012). The GTR+G model was applied to ETS and ITS datasets (White et al. 1990; Baldwin and Markos 1998; Linder et al. 2000; Stanford et al. 2000; Markos and Baldwin 2001), while the TVM+G model was used for trnL-F data (Taberlet et al. 1991). The three sequence regions were subsequently concatenated for phylogenetic analysis. Maximum likelihood (ML) analyses were carried out in IQ-TREE v2 (Minh et al. 2020) with 1,000 bootstrap replicates. The optimal models for gene partitions were determined by ModelFinder (Kalyaanamoorthy et al. 2017).

## ﻿Taxonomic treatment

### ﻿ Aster xuelinii

Taxon classificationPlantaeAsteralesAsteraceae

Z.F.Bai sp. nov.

24A47774-B9DC-59FD-B9FA-5CF492711CC0

urn:lsid:ipni.org:names:77369253-1

Fig. 1

#### Type.

China • Gansu: Hui County, damp stone wall, elev. 800–1100 m, 9 August 2024, *Zengfu Bai & Xuelin Chen 20240043* (holotype NWTC!; isotypes GAUF!).

**Figure 1.**
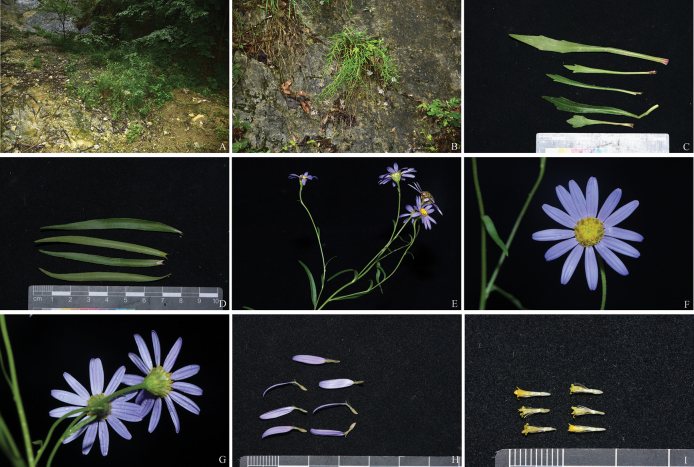
Habitat and morphology of *Aster xuelinii*. A. habitat; B. Habit; C. Basal leaves; D. Cauline leaves; E. Synflorescence; F. Capitulum in frontal view; G. Capitulum in lateral view; H. Disk florets; I. Ray florets.

#### Diagnosis.

This new species resembles *A. dolichopodus* superficially. Both have linear – lanceolate cauline leaves and long pedicels. However, the new species differs by having fewer than 15 ray florets (vs. 19–26), stems that are caespitose, pendent, or rarely ascending, and slender (vs. simple, sometimes caespitose, erect, stout stems). It grows in moist habitats (vs. dry habitats).

#### Description.

Perennial herbs, 30–70 cm tall. Rhizomes short, slightly thick, woody. Stems caespitose, pendent, or rarely ascending, slender, unbranched except for inflorescence, shortly pubescent, with many basal rosette leaves and cauline leaves. Rosette leaves lanceolate, 4–10 × 0.8–1.2 cm, apex acute, base gradually narrowing, strigose, margin serrately one- to two-toothed, petiole 3–5 cm long; lower cauline leaves similar to rosette leaves, sessile, narrowly lanceolate, 4–8 × 0.6–1.4 cm, margin entire or 1-toothed, base gradually narrowing, apex acute, both surfaces of the leaf sparsely scabrous; middle to upper leaves sessile, lanceolate to linear-lanceolate, 3–7 × 0.4–0.7 cm, entire or rarely with a single tooth. Synflorescence leaves lanceolate to linear-lanceolate, 0.5–3 × 0.1–0.4 cm. Capitula 1-6 in terminal corymbiform synflorescences, sometimes solitary; peduncles 6–9 cm long, with linear ca. 2–3.5 mm in diam. bracts. Involucres hemispherical, ca. 5 mm long, 7–15 mm in diameter; phyllaries 5–7-seriate, imbricate, coriaceous, with broadly scarious and shortly ciliate. Outer bracts shorter than inner ones: outer ones lanceolate, only the outermost basally covered with short scabrous hairs; inner ones oblanceolate. True ray 12–14, pale purple, glabrous, 5–9 × 1.2–2 mm; corolla tube 4–6 mm long, apex slightly 2–3-lobed. Disk florets numerous, perfect; tubular yellowish-green, tube 3–4 mm long, glabrous, shallowly 5-lobed; lobes narrowly triangular. Pappus 1-seriate, erect and persistent, off-white in color with minute pubescence adaxially, reaching the base of the corolla lobes or equaling the tube in length. Achenes of both florets similar, narrowly oblong, ca. 2 mm long and 1 mm wide, strigose.

#### Phenology.

Flowering and fruiting from late June to September.

#### Etymology.

This distinctive designation honors the substantial contributions made by Chinese botanist Xuelin Chen to botanical diversity surveys in Gansu Province over many years. Hence, the Chinese name “学林紫菀 (xué lín zĬ wăn)” is suggested.

#### Distribution and habitat.

**Figure 2.**
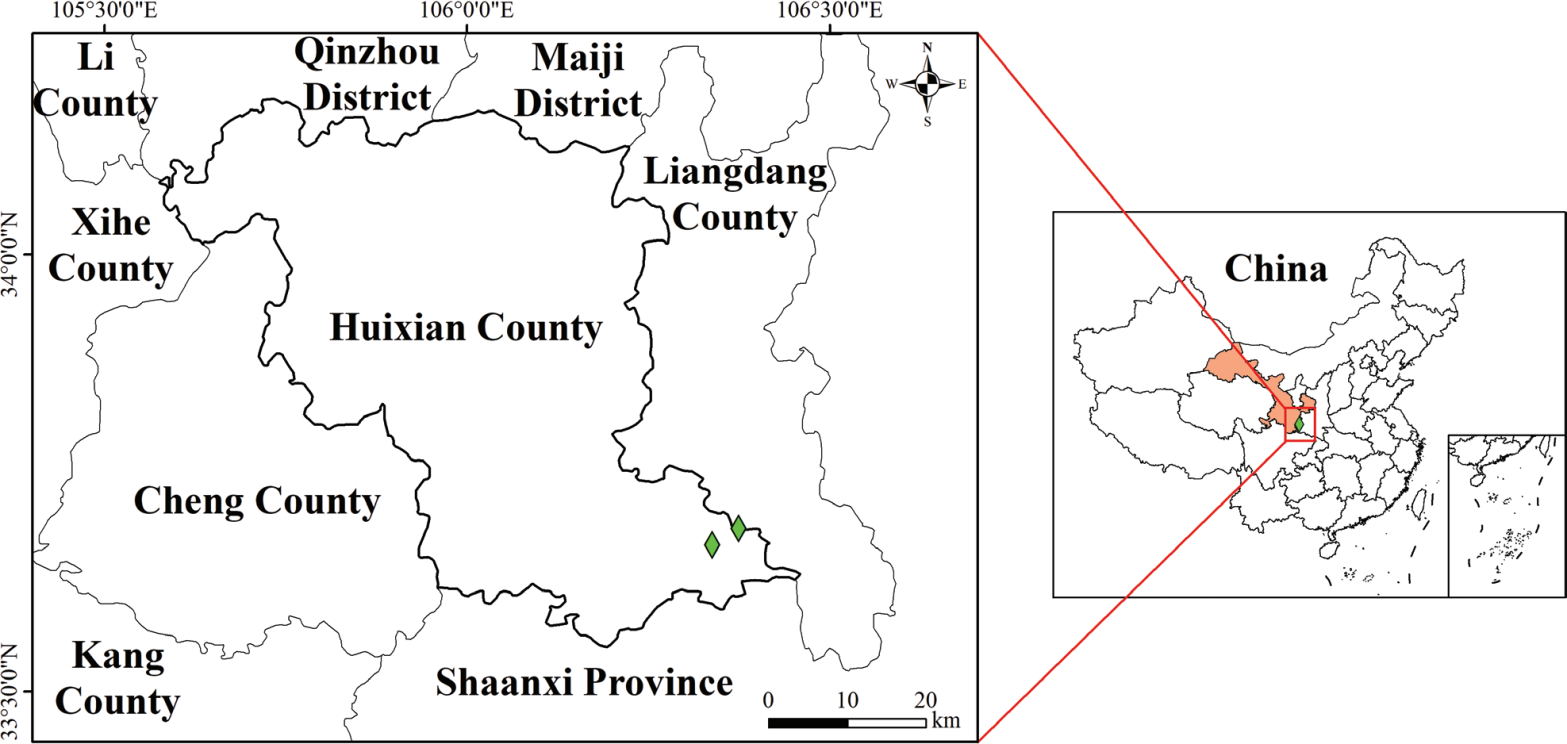
Distribution of *Aster xuelinii*.

*Aster xuelinii* is only known from its type locality, Hui County, Gansu Province, China. This new species grows on moist rock slopes at elevations of 800–1000 meters (Fig. 2).

#### Systematic position.

**Figure 3.**
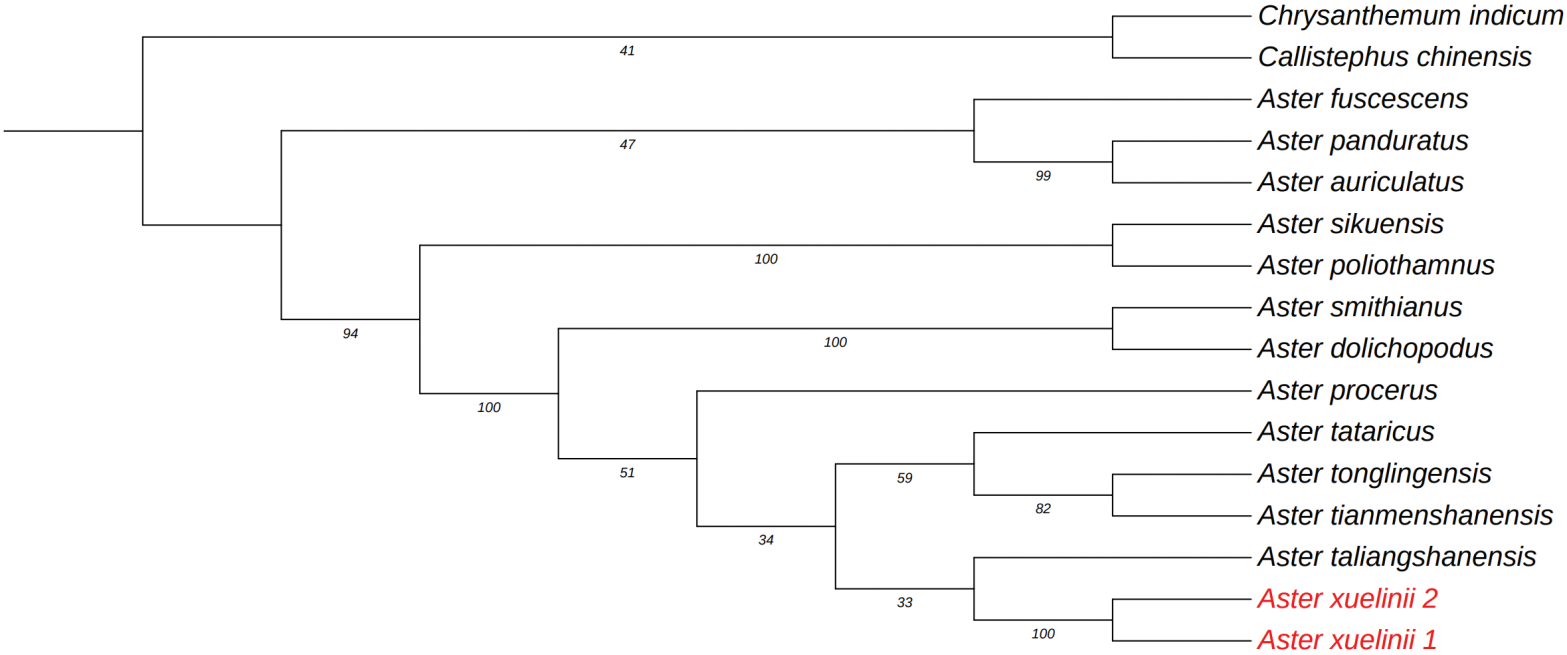
Cladogram of the maximum likelihood (ML) phylogenetic tree of representative *Aster* species. Phylogenetic tree based on combined data (ITS, ETS, and trnL-F), showing the position of *Aster xuelinii*. Numbers at nodes represent maximum likelihood bootstrap percentages. The two specimens of the new species *Aster xuelinii* are marked as red.

Based on molecular phylogenetic analyses, the new species is closely related to *A. taliangshanensis*, and both species are recognized as members of Aster sect. Aster. in Flora of China (Chen et al. 2011) (Fig. 3).

### ﻿Key to distinguish *Aster xuelinii* from the updated key to the species of Aster section Aster.

**Table:** 

1	Woody herbs or subshrubs, open corymbiform synflorescences	***A. smithianus***
–	Perennial herbs, corymbiform synflorescences	**2**
2	Ray florets more than 50	***A. taliangshanensis***
–	Ray florets fewer than 50	**3**
3	Phyllaries 5–7-seriate. Ray florets 12–14, pale purple	***A. xuelinii***
–	Phyllaries 3 or 4-seriate. Ray florets 19-26, light purple to purple	***A. dolichopodus***

## Supplementary Material

XML Treatment for Aster xuelinii

## ﻿Additional information

### Use of AI

No use of AI was reported.

### Data availability

All of the data that support the findings of this study are available in the main text or Supplementary Information.

## Supplementary materials

Supplementary material 1 Voucher information and GenBank accession numbers Data type docx https://binary.pensoft.net/file/1416662This dataset is made available under the Open Database License (http://opendatacommons.org/licenses/odbl/1.0/). The Open Database License (ODbL) is a license agreement intended to allow users to freely share, modify, and use this Dataset while maintaining this same freedom for others, provided that the original source and author(s) are credited.Zengfu Bai, Zhihua Zhang, Guojin Zhang, Ji Zhang
