# Supplementary material for: ﻿ Aster xuelinii (Astereae, Asteraceae), a new species growing on moist rocks from Gansu Province, China
Source: PhytoKeys. 2025 Sep 15;263:17–23. doi: 10.3897/phytokeys.263.166400 (PMC12455216; doi:10.3897/phytokeys.263.166400)
Supplement: Supplementary material 1 — Voucher information and GenBank accession numbers [file phytokeys-263-017_article-166400__-s001.docx]

Table S1 Voucher information and GenBank accession numbers

|  |  | GenBank accessions | | |
| --- | --- | --- | --- | --- |
| Taxon | Voucher or reference | *ITS* | *ETS* | *trnL-F* |
| *Aster auriculatus* Franch. | Li et al. (2012) | JN543754 | JN543755 | JN543756 |
|  | *WPL 0509059* (HNNU) |  |  |  |
| *Aster dolichopodus* Y. Ling | Zhang et al. (2015) | KP313688 | KP313701 | KP313714 |
|  | *Z. X. Fu 70* (PE) |  |  |  |
| *Aster fuscescens* Bur. & Franch. | Li et al. (2012) | JN543751 | JN543752 | JN543753 |
|  | YGS1007021 (HNNU) |  |  |  |
| *Aster panduratus* Nees ex Walp. | Li et al. (2012) | JN543757 | JN543758 | JN543759 |
|  | *WPL 1012067* (HNNU) |  |  |  |
| *Aster poliothamnus* Diels | Li et al. (2012) | JN543763 | JN543764 | JN543765 |
|  | *WPL 0506001* (HNNU) |  |  |  |
| *Aster procerus* Hemsl. | Zhang et al. (2015) | KP313683 | KP313696 | KP313709 |
|  | *Z. X. Fu 693* (PE) |  |  |  |
| *Aster sikuensis* W.W.Sm. & Farrer | Li et al. (2012) | JN543766 | JN543767 | JN543768 |
|  | *WPL 0510025* (HNNU) |  |  |  |
| *Aster smithianus* Hand.-Mazz. | Zhang et al. (2015) | KP313685 | KP313698 | KP313711 |
|  | *Z. X. Fu 143* (PE) |  |  |  |
| *Aster taliangshanensis* Y. Ling | Li et al. (2012) | JN543772 | JN543773 | JN543774 |
|  | *WPL 0607056* (HNNU) |  |  |  |
| *Aster tataricus* L.f. | Li et al. (2012) | JN543748 | JN543749 | JN543750 |
|  | *WPL 0108018* (HNNU) |  |  |  |
| *Aster tianmenshanensis* G. J. Zhang & T. G. Gao | Zhang et al. (2015) | KP313678 | KP313691 | KP313704 |
|  | *C. F. Zhang 2720* (PE) |  |  |  |
| *Aster tonglingensis* G. J. Zhang & T. G. Gao | China, Zhejiang, Wencheng, Mt. Tongling *H. H. Hu 331-1* | MH807116 | MH807115 | MH807117 |
| ***Aster xuelinii* Z.F.Bai** | **Zengfu Bai &Xuelin Chen (2024)** | **PX124783**  **PX124784** | **PX124781**  **PX124782** | **PX124779**  **PX124780** |
|  | ***Zengfu Bai &Xuelin Chen 20240043* (NWTC)** |  |  |  |
| *Callistephus chinensis* (L.) Nees | Li et al. (2012) | JN315931 | JN315955 | JN315907 |
|  | *WPL 0108021* (HNNU) |  |  |  |
| *Chrysanthemum indicum* L. | Li et al. (2012) | JN315940 | JN315964 | JN315916 |
|  | *WPL 1012002* (HNNU) |  |  |  |
| The samples newly sequenced in this study was in bold font. |  |  |  |  |
